# Cytoskeletal Configuration Modulates Mechanically Induced Changes in Mesenchymal Stem Cell Osteogenesis, Morphology, and Stiffness

**DOI:** 10.1038/srep34791

**Published:** 2016-10-06

**Authors:** Suphannee Pongkitwitoon, Gunes Uzer, Janet Rubin, Stefan Judex

**Affiliations:** 1 Department of Biomedical Engineering, Stony Brook University, Stony Brook, NY 11794, USA; 2 Department of Medicine, UNC Chapel Hill, Chapel Hill, NC 27517, USA.

## Abstract

Mesenchymal stem cells (MSC) responding to mechanical cues generated by physical activity is critical for skeletal development and remodeling. Here, we utilized low intensity vibrations (LIV) as a physiologically relevant mechanical signal and hypothesized that the confined cytoskeletal configuration imposed by 2D culture will enable human bone marrow MSCs (hBMSC) to respond more robustly when LIV is applied in-plane (horizontal-LIV) rather than out-of-plane (vertical-LIV). All LIV signals enhanced hBMSC proliferation, osteogenic differentiation, and upregulated genes associated with cytoskeletal structure. The cellular response was more pronounced at higher frequencies (100 Hz vs 30 Hz) and when applied in the horizontal plane. Horizontal but not vertical LIV realigned the cell cytoskeleton, culminating in increased cell stiffness. Our results show that applying very small oscillatory motions within the primary cell attachment plane, rather than perpendicular to it, amplifies the cell’s response to LIV, ostensibly facilitating a more effective transfer of intracellular forces. Transcriptional and structural changes in particular with horizontal LIV, together with the strong frequency dependency of the signal, emphasize the importance of intracellular cytoskeletal configuration in sensing and responding to high-frequency mechanical signals at low intensities.

Mesenchymal stem cells (MSC) residing in bone marrow provide regenerative capacity for bone, replacing and reinforcing the skeleton^1^. The ability of MSC to respond to mechanical cues generated during functional loading is critical for musculoskeletal health. At the cellular level, bone can sense and adapt to daily loading events that induce matrix deformations^2^,^3^, accelerations^4^,^5^, muscle activity^6^,^7^, fluid flow^8^,^9^, or changes in intramedullary pressure^10^,^11^. Loading events do not necessarily have to be large in magnitude to be sensed by cells. In contrast to the low signal frequency of large mechanical forces applied to the skeleton during rigorous exercise, exposure to high-frequency low intensity vibration (LIV) may enhance musculoskeletal function^12^, as LIV may decrease MSC adipogenesis^13^ and adipose tissue^14^, augment bone formation^15^, increase osteogenic lineage output from MSC^16^, or increase muscle size^17^.

Despite their physiologic relevance^18^, little is known about how very small mechanical signals such as LIV are perceived at the cellular level to control output^19^. LIV creates a complex local loading environment that is modulated by many factors including LIV frequency^20^, LIV amplitude^21^, or viscosity of the surrounding fluid^22^. In contrast to high-impact exercise (e.g., jumping) which induces peak bone strains in the extracellular matrix ranging from 1500 to 3500 με^2^,^23^, LIV induces matrix strains of less than 50 με in cortical bone^21^, a deformation unlikely to be large enough to be perceived as a relevant mechanical signal^24^,^25^. Not surprisingly, *in vivo* pre-clinical studies demonstrated that bone’s response to LIV is largely independent of strain generated in the matrix^26^,^27^. Computational studies indicated that LIV (30–100 Hz, 0.1–1 g) can generate considerable fluid shear at trabecular surfaces in contact with bone marrow^22^,^28^. *In vitro* studies, however, could not find a link between LIV induced fluid shear stress magnitude and the cellular response^16^,^29^,^30^.

As an alternative to previously suggested mechanotransduction mechanisms such as matrix deformation or fluid shear, it is possible that cells have mechanisms that allow them to respond to dynamic acceleration rather than matrix deformations per se^5^,^31^. Some aspects of the cellular LIV response, such as increased expression of cytoskeletal proteins^16^, show a positive correlation with the rate of LIV acceleration^31^. Further, LIV-induced signaling specifically requires mechanical coupling between the actin cytoskeleton and nucleus facilitated by the LINC (Linker of Nucleoskeleton and Cytoskeleton) complex^13^, a structural requirement not shared by the signaling events initiated by matrix deformation^32^. These data suggest the possibility that the nucleus, a relatively stiffer and denser organelle mechanically integrated into the cytoskeleton^29^, may play a role in LIV-signaling through transmitting acceleration to the cell cytoskeleton from inside the cell and thus generating internal cellular stresses through motions relative to the cell cytoskeleton and cell membrane. Even though the sub-cellular localization of these LIV-induced signaling events remains to be uncovered, LINC complexes may also play a role in activating force-responsive signaling event within the nucleus via exerting forces through their connection with the cytoskeleton^33^.

If indeed LIV activates mechano-signaling pathways through the connectivity between nucleus and cell cytoskeleton, the efficacy of LIV should be dependent on structural cell configuration as both cytoskeletal pre-stress^34^, controlling the degree of mechanical coupling between nucleus and actin cytoskeleton^35^, and spatial configuration of actin filaments^36^ may play a role in transmittal of forces. Thus, in a 2D cell culture system that confines the cell architecture into a single plane with a forced apical-basal polarity^37^, it is possible that the application of horizontal accelerations is perceived differently by the cell from vertical accelerations as cell configurational differences relative to the principal motion axis will generate different cellular stresses.

To answer this question, we applied LIV in 2D cell culture either horizontally or vertically with respect to the plane of cell attachment and quantified outcome variables including proliferation, differentiation, gene expression, and cytoskeletal structure/stiffness of human bone marrow MSC (hBMSC). We hypothesized that cells would be more responsive to horizontal vibrations as they have the potential to engage the nuclear-cytoskeletal structure more effectively in its 2D horizontal plane. We also hypothesized that the relative importance of LIV amplitude and frequency in hBMSC’s mechanoresponse would be independent of LIV direction.

## Results

### Overview of Experimental Design

To understand if altering the direction in which vibrations are applied plays a role in controlling MSC differentiation and cytoskeletal actin structure and whether LIV amplitude and frequency interact with LIV direction, we applied LIV twice daily for 20 min in either horizontal (h-LIV) or vertical (v-LIV) direction at frequencies of 30 Hz or 100 Hz with acceleration magnitudes of 0.15 g or 1 g. Changes in proliferation (Day 3), ALP activity (Days 1 & 7) and calcium deposition (Days 7 & 14) were assessed.

As both the cytoskeleton and nucleo-cytoskeletal connections are involved in specific aspects of LIV mechanosensing^13^,^38^, gene expression profiles of cytoskeletal, nucleo-skeletal, and cell-cell adhesion regulatory proteins were determined via PCR arrays on Days 1, 7, and 14. Additionally, transcriptional levels of genes selected for their role in differentiation were quantified on Day 1. To elucidate morphologic and mechanical adaptations to the distinct LIV signals, the orientation of F-actin was quantified via two-photon confocal microscopy and differences in cellular stiffness between groups were quantified via atomic force microscopy (Day 1). A sample size of n = 6, run as triplicates, was used throughout unless noted otherwise.

### hBMSC proliferation

**Figure 1:**
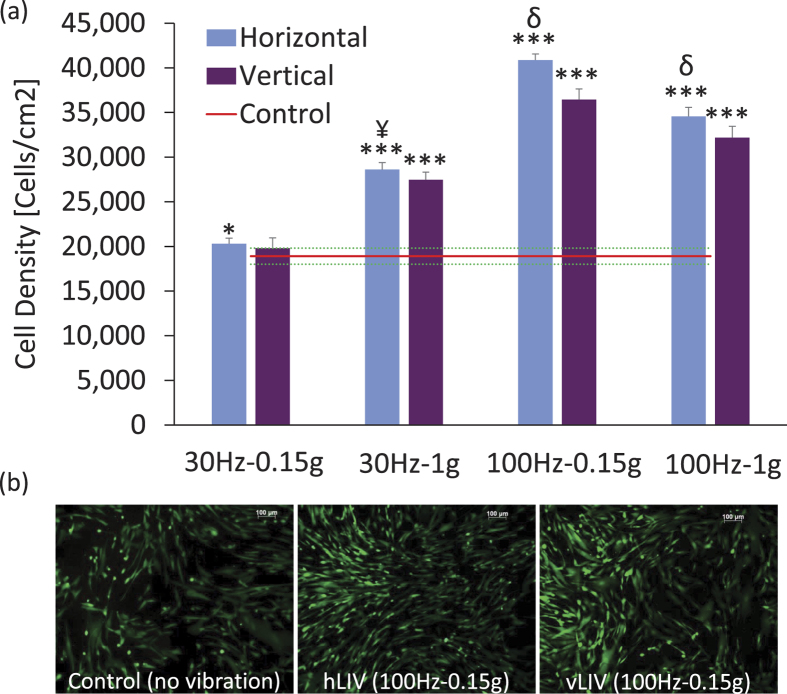
(**a**) Mesenchymal stem cell (MSC) density of cell dishes subjected to 30 Hz-0.15 g (30–0.15), 30 Hz-1 g (30–1), 100 Hz-0.15 g (100–0.15), or 100 Hz-1 g (100–1) LIV in both horizontal and vertical directions. The red line represents the mean value for non-LIV control MSCs (±SD). Cell density was significantly greater in all LIV groups than in controls. (**b**) Images showing differences in cell density between control, horizontal 100 Hz-0.15 g LIV and vertical 100 Hz-0.15 g LIV groups at Day 3. *p < 0.05; **p < 0.01; ***p < 0.001, against Control. ^¥^*p* < 0.05; ^†^*p* < 0.01; ^δ^*p* < 0.001, Horizontal vs Vertical.

Fluorescent images of calcein stained cells showed no differences in viable cells between LIV and non-LIV conditions (not shown). On Day 3 of the experiment, cell density was greater (p < 0.001) in all LIV groups when compared to non-LIV controls (Fig. 1a,b). For both horizontal and vertical LIV directions, the 100 Hz-0.15 g signal had the greatest effect on proliferation (Fig. 1a). When directly comparing h-LIV to v-LIV, cell density was greater for h-LIV than v-LIV for the 100 Hz-0.15 g (12%, p = 0.02) and 100 Hz-1 g (7%, p = 0.03) groups but not for the 30 Hz groups. Cell numbers in dishes exposed to 100 Hz signals had a 42 ± 8% greater (p < 0.001) cell number than those exposed to 30 Hz signals (averaged across loading directions and accelerations).

### hBMSC osteogenic commitment

To evaluate the effects of LIV direction on osteogenic differentiation of hBMSCs, we measured Runx2 and ALPL mRNA activity (Day 1), ALP activity (Day 1 & 7), and Ca^2+^ deposition (Days 7 & 14). In all LIV groups, mRNA levels for ALPL and Runx2 were significantly elevated compared to non-LIV controls (Table 1). Compared to non-LIV controls, h-LIV elicited higher gene expression levels, on average, for ALPL (18 ± 4.6%, p < 0.001) and Runx2 (21 ± 3.2%, p < 0.001). The greatest upregulation was observed after horizontal 100 Hz-0.15 g exposure for both ALPL (22%, p < 0.001) and Runx2 (24%, p < 0.001) (Table 1).

**Figure 2:**
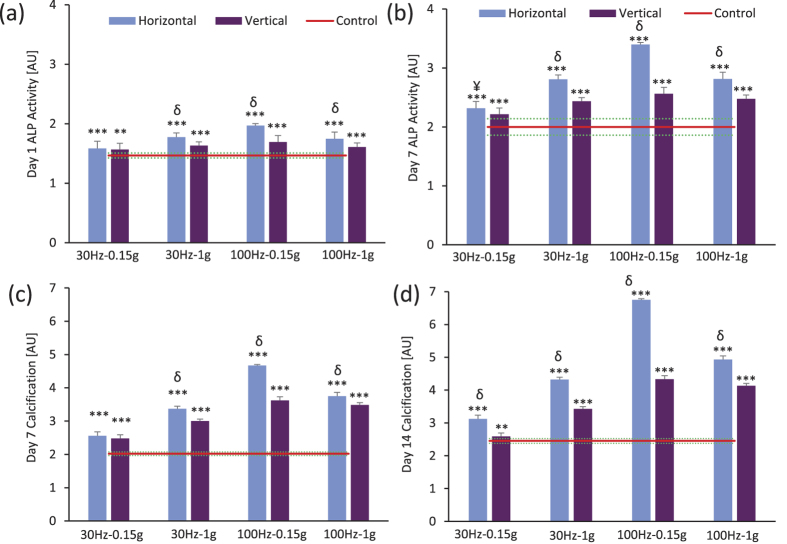
Levels of (**a,b**) alkaline phosphatase activity (Days 1&7) and (**c,d**) calcification (Days 7 & 14) pertaining to the four LIV conditions. The red line represents the mean value for non-LIV MSCs (control). Both alkaline phosphatase activity and mineralization was significantly greater in all LIV groups than in controls. *p < 0.05; **p < 0.01; ***p < 0.001, against Control. ^¥^*p* < 0.05; ^†^*p* < 0.01; ^δ^*p* < 0.001, Horizontal vs Vertical.

On Day 1 (Fig. 2a) & Day 7 (Fig. 2b), alkaline phosphatase activity was significantly elevated in all LIV treated groups when compared to non-LIV controls. Differences between h-LIV and v-LIV treatments were greater on Day 7 than on Day 1. The average difference between h-LIV and v-LIV was 16% (p < 0.001) on Day 7 (Fig. 2b) and 9% (p < 0.001) on Day 1 (Fig. 2a) with 100 Hz-0.15 g showing the greatest difference (32%, p < 0.001, Fig. 2a,b). This specific LIV combination also had the greatest elevations in ALP compared to other frequency/acceleration combinations (p < 0.001).

Calcium deposition (mineralization), as quantified by alizarin red staining intensity, was greater in all LIV groups than in non-LIV controls on Days 7 and 14 (Fig. 2c,d). Consistent with the hypothesis that the directional application of LIV with respect to cell attachment plane can differentially modulate the osteogenic commitment of hBMSC, differences in calcium deposition between h-LIV and v-LIV groups were significant for both 30 Hz and 100 Hz frequencies measured on Days 7 (Fig. 2c, p < 0.001) & 14 (Fig. 2d, p < 0.001). The average difference between h-LIV and v-LIV was 32% (Fig. 2d, p < 0.001) on Day 14 and 14% (Fig. 2d, p < 0.001) on Day 7 with 100 Hz-0.15 g showing the greatest difference (32%, p < 0.001, Fig. 2c,d). LIV direction had a significant effect on hBMSC differentiation; on both Days 7 & 14, the difference in calcification between h-LIV and v-LIV groups was largest in those cultures exposed to 100 Hz-0.15 (difference of 56% on Day 14, p < 0.001).

### Higher frequency enhanced gene expression induced by both horizontal and vertical LIV

**Figure 3:**
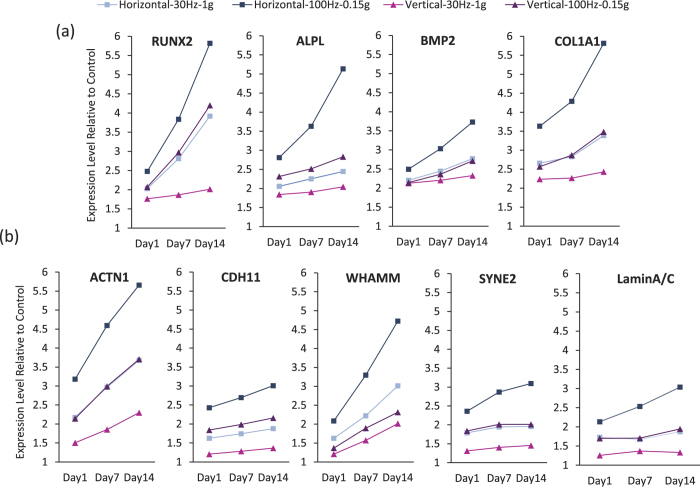
Normalized transcriptional activity of (**a**) genes associated with osteogenic differentiation and matrix maturation and (**b**) genes associated with cytoskeletal and nucleoskeletal organization that were up-regulated by more than 3-fold over non-LIV controls after exposure to either horizontal or vertical LIV at 30 Hz-1 g or 100 Hz-0.15 g on Days 1, 7, and 14.

Gene arrays using the most effective signal combinations at 30 Hz (30 Hz-1 g) and 100 Hz (100 Hz-0.15 g) showed that genes associated with osteogenic differentiation and matrix maturation - ALPL, BMP2, COL1A1, and RUNX2 - were strongly (>3-fold) upregulated by both h-LIV and v-LIV (Fig. 3a). Further, genes associated with cytoskeletal and nucleoskeletal organization, ACTN1, CDH11, WHAMM SYNE2 and LaminA/C, were upregulated by more than 3-fold for at least one LIV intervention (Fig. 3b). For all upregulated genes, differences in mRNA transcriptional activity between LIV groups and controls became larger as experimental duration progressed from 1d to 7d to 14d, suggesting an accumulative effect of LIV (Fig. 3a,b). For all eight genes in this group of greater than 3-fold expression changes and for all three time points considered, 100 Hz LIV signals induced 39 ± 11% (p < 0.001) greater gene expression than 30 Hz signals, demonstrating a frequency-dependent enhancement of hBMSC gene expression.

For individual genes in this group, α-actinin (ACTN1), a crosslinking protein known to play a role in stabilizing actin stress fibers^39^, was more highly upregulated (averaged across all time points) by h-LIV than v-LIV at 100 Hz (52%) and 30 Hz (56%). CDH11, a protein that tethers stress fibers to cadherins at cell junctions^40^, had 35% (100 Hz) and 36% (30 Hz) greater transcriptional levels with h-LIV. Gene expression of Nesprin-2 (SYNE2), the actin-binding element of the LINC complex^41^ and LaminA/C, a LINC anchoring nuclear matrix protein known to scale with nuclear stiffness^42^, were upregulated by >3-fold only in the horizontal 100 Hz-0.15 g group on Day 14. Averaged across the 3 time points, h-LIV gave rise to greater mRNA activity than v-LIV at both 100 Hz (SYNE2: 41%, LaminA/C: 43%) and 30 Hz (SYNE2: 37% LaminA/C: 33%). Further, the WASP homologue associated with actin, membranes, and microtubules (WHAMM), a nucleation-promoting protein regulating Arp 2/3 complex branched remodeling of both actin and microtubule cytoskeletons^43^, had 21 ± 0.4% greater expression levels with h-LIV than v-LIV at 100 Hz and 15 ± 0.1% at 30 Hz (Fig. 3b).

For genes upregulated between 2.5 and 3-fold (Table 2), h-LIV promoted 22% greater transcription than v-LIV when averaged over all genes on Days 1 (18%), 7 (21%), and 14 (27%). On Day 14, horizontal 100 Hz-0.15 g LIV produced transcriptional levels that were 41% greater than the average of the other three experimental groups. CD44^44^, BMP-4^45^, and ATF-4^46^ have all been associated with osteoblastogenesis and their up-regulation, particularly in the horizontal 100 Hz-0.15 g group at Day 14, highlight the propensity of this specific LIV signal to induce differentiation in hBMSC. Both the upregulation of Rho GTPase CDC42 (consistent with previous data^16^) and SUN-2, the anchoring element of Nesprin-2^47^, may point towards LIV enhancing cellular structure.

LIV also elicited between 2-2.5-fold upregulation in 28 other genes known to play a role in matrix maturation, osteogenesis and cytoskeletal organization (Table 3). Among the 28 genes that were upregulated, h-LIV produced, on average, 16% greater transcriptional levels than v-LIV at Day 14. Across genes, the transcriptional increase in horizontal 100 Hz-0.15 g was 26% greater than the average of the three other groups.

### LIV changes in cell stiffness and orientation

To test whether the LIV induced changes in cytoskeletal proteins remodeled the cytoskeletal structure and its mechanical properties, we used the LIV frequency and acceleration that was consistently most effective in eliciting cellular and molecular changes in both horizontal and vertical directions (100 Hz-0.15 g).

**Figure 4:**
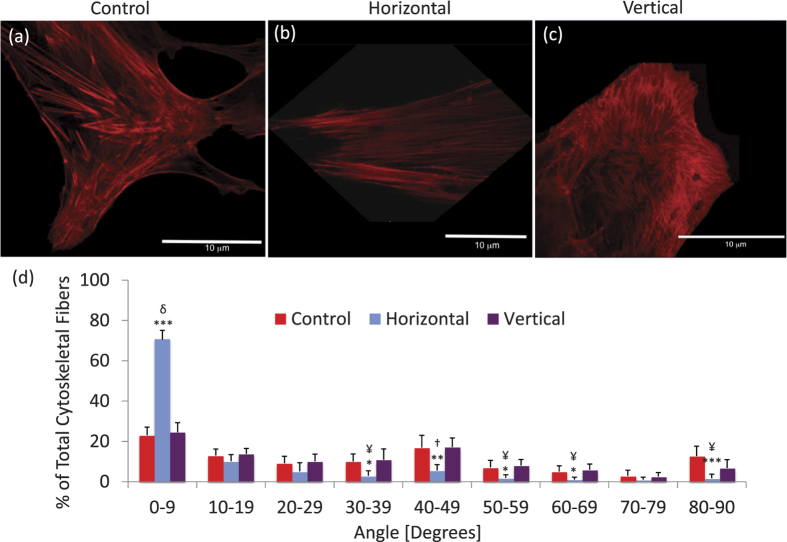
Two-photon confocal microscopic fluorescent images of cytoskeletal fibers after exposure to (**a**) non-LIV, (**b**) horizontal LIV (100 Hz-0.15 g), or (**c**) vertical LIV (100 Hz-0.15 g). (**d**) Histogram of *in-plane* (x–y) cytoskeletal orientation of actin with respect to the direction in which horizontal vibration was applied. 0 is the direction parallel to the horizontal vibration direction, 90 is perpendicular to the horizontal vibration direction. The x-axis presents the magnitude of range of angles; 0–9 = −9–(+9) degrees, 10–19 = −10–(−19) & 10–19 degrees, etc. *p < 0.05; **p < 0.01; ***p < 0.001, against Control. ^¥^*p* < 0.05; ^†^*p* < 0.01; ^δ^*p* < 0.001, Horizontal vs Vertical.

Two-photon confocal microscopy was used to visualize cytoskeletal orientation. Visualization of F-actin (Fig. 4a,c, Fig. S1) showed that hBMSCs not subjected to LIV exhibited randomly oriented stress fibers (Fig. 4a). h-LIV realigned stress fibers such that their longitudinal axis coincided with the direction of the applied vibration (Fig. 4b). In contrast, no change in stress fiber orientation was observed with vertical LIV (Fig. 4c). Consistent with the increases in Nesprin-2 and Whamm gene expression, 70% (p < 0.001) of visualized F-actin fibers were aligned within 10 degrees of the vibration axis in h-LIV treated cells.

**Figure 5:**
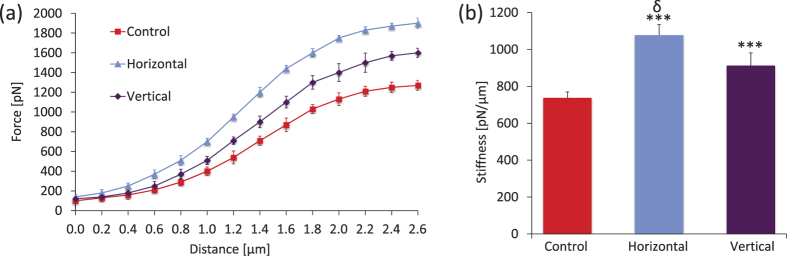
(**a**) Averaged force-displacement curves for cells under control conditions, horizontal LIV (100 Hz-0.15 g), or vertical LIV (100 Hz-0.15 g). Each data point presents the mean ± SD of six samples. **(b)** Cell stiffness, defined as the slope of the linear part of the force-displacement curve, was significantly greater in vibrated than in control cells with horizontal vibration initiating the greatest difference in cell stiffness. *p < 0.05; **p < 0.01; ***p < 0.001, against Control. ^¥^*p* < 0.05; ^†^*p* < 0.01; ^δ^*p* < 0.001, Horizontal vs Vertical.

AFM measurements on Day 1 immediately after LIV showed that compared to cellular stiffness in non-vibrated hBMSC, stiffness was 46% greater (p < 0.001) in h-LIV cells and 24% greater (p < 0.001) in v-LIV cells (Fig. 5a,b). Further, h-LIV hBMSCs were 18% (p < 0.001) stiffer than v-LIV cells.

### CDH11, ACTN1, Nesprin-2 and LaminA/C

To further test if the enhanced alignment with vibration direction and altered stiffness was mirrored in LIV-responsive genes on Day 1, we used flow cytometry and PCR to probe increases in Cdh11 (as a measure of intra-cellular connectivity at cell edges), Actn1 (cytoskeletal connectivity) and Nesprin-2 (nucleo-cytoskeletal connectivity) protein and transcript levels. LaminA/C was only tested at the transcript level due to sample availability. We found that compared to non-vibrated controls, both h-LIV (Cdh11: 33%, Actn1: 55%, Syne2: 38%; all p < 0.001) and v-LIV (Cdh11: 9%, Actn1: 24%, Syne2: 18%; all p < 0.001) elicited increases in protein levels (Table 4). Further, protein levels in h-LIV treated groups were elevated to a greater degree than in v-LIV groups (Cdh11: 21%, Actn1: 25%, Syne2: 17%; all p < 0.001). Similarly, compared to non-vibrated controls, both h-LIV (Cdh11: 264%, Actn1: 331%, Syne2: 253%; all p < 0.001) and v-LIV (Cdh11: 187%, Actn1: 210%, Syne2: 167%; all p < 0.001) elicited increased gene expression levels (Table 4). Gene expression levels in h-LIV treated groups were elevated to a greater degree than in v-LIV groups (Cdh11: 141%, Actn1: 157%, Syne2: 150%, LaminA/C: 156%; all p < 0.001). The additional augmentation in the expression levels of these proteins suggests that horizontal LIV may generate greater intracellular forces.

### Relative role of LIV frequency and acceleration

Since the loading environment generated by LIV is an outcome of the combination of both LIV frequency and LIV acceleration magnitude, we tested for the relative contributions and possible interactions of these two factors. During early cell proliferation experiments, two-way ANOVA showed a significant interaction between acceleration magnitude and frequency (p < 0.001, across LIV directions). LIV frequency accounted for 59% of the variability in cell density (p < 0.001) while LIV acceleration accounted for only 1% of the total variability (p = 0.02). Later time points also showed significant interaction between acceleration magnitude and frequency in both ALP activity (p < 0.001) as well as mineralization (p < 0.001). While LIV frequency accounted for 27% (p < 0.001) of variability in ALP activity and 49% (p < 0.001) in mineralization, contribution of acceleration magnitude to the total variance was less than 0.1% (NS).

## Discussion

LIV influences fate selection of MSCs by initiating mechanosensitive signaling pathways in cells, ultimately leading to improvements in musculoskeletal outcomes and adiposity^19^. Here, we asked if the directional application of LIV with respect to the plane of cell attachment differentially modulates MSC differentiation and cytoskeletal remodeling. We found that when applied parallel to the cell attachment plane (h-LIV), LIV was more effective at promoting MSC osteoblastogenesis, ALP activity and mineralization compared to the application of vertical LIV (v-LIV). Accompanying these changes, gene expression of cytoskeletal and nucleoskeletal regulatory genes was increased to a greater extent by h-LIV than v-LIV. Consistent with evidence of increased architectural components, AFM measurements revealed that cell stiffness in cells exposed to h-LIV was greater than in those exposed to v-LIV. Irrespective of vibrations direction, 100 Hz LIV combinations were more potent signals than 30 Hz combinations, in particular when combined with the lowest LIV magnitude used here (0.15 g). In support of a greater role for LIV frequency over acceleration in orchestrating the cellular LIV response, there was a significant interaction between LIV frequency and acceleration magnitude with a relatively small role for acceleration magnitude. Together, our data implicate a critical role for the structural configuration of the cytoskeleton for sensing and responding to low-level oscillatory mechanical signals.

A conceivable modulator of the cellular response to LIV may be the induced fluid shear stress^22^,^28^. As h-LIV causes two orders of magnitude greater fluid shear stresses than v-LIV^13^,^30^, we cannot exclude the possibility that fluid shear stress may have contributed to the greater magnitude of the h-LIV response observed here. However, not only did we previously fail to find any direct effect of fluid shear stress on the h-LIV response^16^,^29^, our current data also reject the hypothesis of fluid flow driving the LIV response as the signal combination of 100 Hz-0.15 g, inducing the lowest fluid shear (<0.02 Pa)^30^, generated greater responses. Further, increasing the level of fluid shear stress (by increasing the acceleration from 0.15 g to 1 g^30^) did not potentiate the measured outcomes. Thus, it is unlikely that fluid shear should be considered in the interpretation of our findings.

The cell nucleus is mechanically integrated into the cell architecture^48^ and participates in mechanosensing and fate selection of MSCs^49^. The physical coupling between the nucleus and cytoskeleton is facilitated via the LINC complex^32^,^50^, comprising Nesprin and Sun proteins^50^ that enable the sensing of LIV^13^. Application of LIV activates RhoA signaling to initiate cytoskeletal reorganization, including formation of new focal adhesions and possibly increases the nucleo-cytoskeletal connections at the perinuclear domain^13^. In this regard, formins are increasingly being recognized for playing a role in remodeling of perinuclear architecture. FHOD1 formin^51^, for example, has recently been shown to interact with Nesprin-2 and may be important for nuclear positioning and maintaining coupling between the nucleus and cytoskeleton. Further, LINC complexes may also be involved in formin mediated perinuclear and intranuclear actin dynamics^52^,^53^, suggesting that formins play a yet to be determined role in LINC mediated LIV mechanotransduction. Our data demonstrated that the expression levels of Nesprin-2, the actin-binding element of the LINC complex as well as the nuclear scaffolding protein LaminA/C experienced the greatest upregulation at Day 14 when exposed to horizontal 100 Hz-0.15 g LIV. As MSC mechanosensitivity increases with LIV bouts^13^, the temporal increase in Nesprin-2 and LaminA/C expression may suggest an increased nucleo-cytoskeletal connectivity that may, at least in part, contribute to a more potent MSC mechanoresponse with long-term LIV treatment.

We have previously shown that cellular stimulation with LIV activates similar signaling pathways when compared to stretching cells at low frequency (mechanical strain)^13^. The critical difference between LIV (inside-inside) and mechanical strain (outside-inside) appears to be the requirement of LINC connections rather than the signaling pathways activated. More specifically, overexpression of Nesprin KASH domain or siRNA against Sun1/2 does not block strain induced FAK activity^13^ but inhibits LIV signaling. Conversely, activation of Akt in response to strain requires Fyn activity^54^ while LIV does not utilize this pathway to activate Akt^13^. These findings suggest that both LIV and strain activate similar signaling pathways. Further, the LINC requirement of LIV-induced signaling may suggest that intra-cellular connectivity plays a role in activating conventional signaling pathways in response to LIV.

Consistent with previous data^16^ was the upregulation of the WAS family of genes with h-LIV. Whamm, a protein involved in remodeling of both actin and microtubule cytoskeletons^43^, was consistently upregulated in all h-LIV groups. As Nesprin is required to maintain strain-induced nuclear alignment^55^, the upregulation of Whamm and Nesprin-2 suggests that the direction of the LIV signal may also play a role in nuclear orientation and its cytoskeletal connection. Moreover, expression levels of Sun2, the Nesprin binding element of LINC complex^47^, were upregulated. As LINC complexes are important for signaling pathways such as βcatenin^56^, it will be important to consider in future studies whether different structural adaptations to LIV direction contribute to how cells respond to subsequent mechanical or biochemical cues. Further, we and others have previously shown defects in cell migration and alignment in response to mechanical challenges like strain using LINC deficient cells *in vitro*^13^,^32^,^57^. When using siRNA against Sun1/2 or using the DN-Nesprin KASH domain, LIV cannot activate RhoA. It may therefore be possible that limiting LINC connectivity may also eliminate the h-LIV induced cell orientation demonstrated here. Further investigation of the effects of LINC deficiency on the LIV-induced cytoskeletal realignment will provide important clues towards elucidating LIV mechanotransduction.

Alpha-actinin supports higher-order cytoskeletal stress fiber formation^39^. CDH11 directs adherent junction formations during cell-cell bridging and can interact with structural proteins including α-actinin^40^. Considering that both ACTN1 and CDH11 were more highly upregulated in h-LIV groups in which cytoskeletal orientation aligned with the loading axis, the concomitant increase in these two proteins by h-LIV may suggest a coordinated effort towards regulating cellular alignment and cell-to-cell interaction. Interestingly, SUN2 has recently been implicated in mediating cell-cell contact in keratinocytes^58^. As h-LIV enhances gap junctional communication in MLO-Y4 osteocyte like cells^29^, the enhanced expression in CDH11 and SUN2 in h-LIV groups may point towards an enhancement in LIV induced cell-to-cell interaction. If true, then cells exposed to h-LIV may achieve their more highly organized cellular architecture through the upregulation of these structural genes.

Identification of cellular elements involved in sensing and responding to extremely low levels of mechanical signals may prove critical towards developing effective mechanically based musculoskeletal treatments without side-effects. Our work here focusing on cellular and molecular responses when exposed to low intensity vibrations emphasizes the importance of the cell’s cytoskeletal and nucleo-cytoskeletal elements in transforming LIV signals into a biological response in human mesenchymal stem cells.

## Methods

### Cell culture

Commercially available human hBMSC (25yr old African American female, Thermo Fisher Scientific) were cultured in alpha-MEM without phenol red containing 7.5% heat-inactivated fetal bovine serum (Thermo Fisher Scientific), 5 μM L-glutamine and specific growth factors of recombinant 15 nM IGF-1 and 125 pM FGF-β. To protect cells from changing environments, 1 mM DTT (D1532, Thermo Fisher Scientific) dissolved in 0.1% BSA (A2058, Sigma) was added to the stock solution of growth factors. Since hBMSC are sensitive to changes in pH and %CO_2_, we added 25 mM HEPES to preserve a pH of 7.4 and 1% penicillin/streptomycin to protect against infection.

For all experiments other than proliferation, hBMSCs were plated at a cell density of 18,000 cells/cm^2^ in 24 wells/plate, two days prior to the experiment. Two days after the first inoculation, hBMSCs were induced with osteogenic media containing 100 nM dexamethasone, 10 mM β-glycerol phosphate, and 0.05 mM L-ascorbic acid-2-phosphate. LIV treatment was commenced immediately following osteogenic induction. The osteogenic medium was changed every other day throughout Day 14. For measuring cell proliferation, we plated hBMSCs at 7,500 cells/cm^2^ on the day prior to the experiment.

### Application of low intensity vibration (LIV)

hBMSCs were vibrated at 30 Hz or 100 Hz using 0.15 g or 1 g accelerations in either horizontal or vertical directions. LIV frequencies and accelerations were selected based on our previous studies^16^,^21^. LIV was applied at room temperature. Control cells were handled identical to LIV cells using 0 Hz and 0 g as LIV parameters. Cells were vibrated for 20 min twice a day separated by a 2 h rest period^59^. After LIV treatment, cells were returned to the incubator.

### Cell proliferation assay

For measuring cell proliferation, we plated hBMSCs at 7,500 cells/cm^2^ on the day prior to the experiment. Cell density (cells/cm^2^) was determined immediately after the second vibration as an indicator of proliferation. A standard spectrophotometric MTS assay was used according to the manufacturer’s instructions (XTT Assay, ACTT).

### Alkaline phosphatase

To measure alkaline phosphatase activity, hBMSCs were rinsed with sterilized distilled water before adding 75 μl of 0.5 mM p-NP. After 1 h, 0.2 M NaOH was added to stop the reaction. The colorimetric assay was performed on Days 1 & 7.

### Cellular calcification

After fixing cells with ice-cold 100% ethanol, cells were rinsed with sterilized distilled ice-cold water. A 40 mM Alizarin Red S (A5533, Sigma) solution was added to the fixed cells and incubated for 45 min at room temperature in the dark. Subsequently, samples were washed with sd-H_2_O before incubation with dPBS for 15 min. To quantify calcification, alizarin red stained cells were de-stained with 10% cetylpyridinium chloride (C0732, Sigma) for 15 min at room temperature in the dark. Fluorescence absorbance at 562 nm was measured on Days 7 & 14.

### RT-PCR

Genes on the 96-gene array were selected based on their involvement in cytoskeletal remodeling, nucleoskeletal organization, and osteogenic commitment (Supplementary Table 1). Cells were cultured in osteogenic media. For this gene array experiment, the signal combinations found to be most effective at 30 Hz (30 Hz-1 g) and 100 Hz (100 Hz-0.15 g) were used. Cells were vibrated for 14d and gene expression levels were quantified on Days 1, 7, and 14. At each time point, cells were lysed with TRIzol (Thermo Fisher Scientific) and RNA was extracted with RNeasy (Qiagen). NanoDrop (ND-100 V3.3.0) quantified RNA, cDNA, and nucleic acid concentration and quality. mRNA was reverse transcribed (Thermo Fisher Scientific) and equivalent amounts of cDNA from each of the six samples within any given group was pooled into one tube and exposed to a custom made 96-gene PCR array (RT^2^ Profiler, Qiagen). Transcription levels were quantified with a standard TaqMan protocol according to manufacturer’s instructions (Thermo Fischer Scientific). LIV induced fold-changes of each gene were determined with the ΔΔCt method^60^ and expressed as percentage of controls with GAPDH levels as referent. LIV induced changes in gene expression were stratified into those that were at least 3-fold greater (or smaller) than in non-vibrated controls, 2.5–2.9 fold greater/smaller, 2.0–2.4 fold greater/smaller, or less than 2-fold greater/smaller.

We also used RT-PCT to determine transcriptional levels of alkaline phosphatase (ALP), Runt-related transcription factor 2 (RUNX2), Nesprin-2 (SYNE2), and Cadherin-1 (CDH1) on Day 1. For this step, cDNA was not pooled across samples and a sample size of n = 6 was used. A standard Taqman protocol was applied.

### Flow cytometry

After the second LIV treatment on Day 1, attached cells were lifted off the surface with 0.05% trypsin/EDTA. Cells were stained against α-actinin (ACTN1, sc-17829, Santa Cruz Biotechnology), Nesprin-2 antibody (SYNE-2, ab57397, Abcam) and Cdh11 (sc-1502, Santa Cruz Biotechnology). For data acquisition, the % gated cell population positive for a specific immunofluorescence was set at least 10,000 events and analyzed in FlowJo.

### Two-photon confocal microscopy

To quantify the degree of cytoskeletal orientation, cytoskeletal F-actin bundles were labeled with Rhodamine Phalloidin (R415, Thermo Scientific). Briefly, cells were permeated by 0.2% Triton for 5 min before the nucleus was immuno-fluorescently labeled with DAPI specific to nuclear DNA and nucleic acid (4′, 6-diamidino-2-phenylindole/2HCl). Cells were further incubated overnight with approximately 6.6 μM Phalloidin at 4 °C. Images were taken at 63× (water lens) using a Zeiss two-photon laser scanning confocal microscope (LMS510 META NLO).

The total stack-height of the acquired images was about 100 μm using an interval of 4.2 μm for each acquisition. The cytoskeletal F-actin bundles were identified by spatial interpolation of the basal and apical surfaces and the image was captured. Six images per well were analyzed, capturing at least 3–6 cells per image. After conversion to binary images, all slices of the 3D tomography were collapsed into a single horizontal 2D plane (NIH ImageJ). Edge detection identified individual cytoskeletal fibers (>100 per cell). Fiber orientation in the vertical plane was not performed because of insufficient resolution in this direction. The reference axis used to calculate the orientation of cytoskeletal fibers coincided with the direction of h-LIV application in all three groups. In increments of ±10 degrees, the percentage of fibers oriented within a given range of angles from the reference axis was quantified for all cells. At least 18 cells per well were analyzed and values were averaged across cells to preserve the sample size of n = 6 per group.

### Atomic Force Microscopy

Cytoskeletal stiffness of control and vibrated cells was estimated by atomic force microscopy (Nanoscope III MultiMode, Digital Instruments, Veeco) with a fluid holder (DI 3100, Veeco). Drive amplitude (mV) and lateral deflection amplitude of the cantilever (mV, ΔX) were converted to force (pN) and vertical displacement (μm). For imaging the samples, the photodetector was set at 3–4 V (negative set-point at 0.5–1 V) with a scan rate of 203 Hz and a resolution of 1.5 μm. Calibration was performed in Hank’s balanced solution as described previously^60^. Before each measurement, the standard V-shaped cantilever (200 μm) and tip (typical radius of the apex ~20 nm) were sanitized by UV shortwave for >2 min.

To commence the measurement, the tip was guided over a cell via a laser system. The cytoskeleton inside the cell was located (at around half the total height of the cell) and the tip was placed over the center of an actin fiber. For stiffness measurements, vertical displacement of the tip was approximately 3 μm. The ratio of the force to the vertical displacement in the linear portion of the force-displacement curve was calculated as stiffness (pN/μm). Three measurements in each of the 6 wells per group were made and averaged.

### Statistical Analysis

Results were presented as mean ± SD. With the exception of the gene array, two-photon microscopy, and AFM assays, all experiments were run in triplicates and data were averaged to maintain n = 6 for statistics. Differences between groups were identified by one-way analysis of variance (ANOVA) followed by Student-Newman-Keul (SNK) post-hoc tests. Main effects of, and interactions between, frequency, acceleration, and/or direction were evaluated using two-way and three-way ANOVA. p < 0.05 was considered significant.

## Additional Information

**How to cite this article**: Pongkitwitoon, S. *et al*. Cytoskeletal Configuration Modulates Mechanically Induced Changes in Mesenchymal Stem Cell Osteogenesis, Morphology, and Stiffness. *Sci. Rep.* **6**, 34791; doi: 10.1038/srep34791 (2016).

## Supplementary Material

Supplementary Information

## Figures and Tables

**Table 1:** Normalized expression of alkaline phosphatase (ALP), Runt-related transcription factor 2 (RUNX2), examined on Day 1 via quantitative PCR.

Gene	Control	Horizontal				Vertical			
	CT	30–0.15	30–1	100–0.15	100–1	30–0.15	30–1	100–0.15	100–1
ALPL	1.0 ± 0.06	2.1 ± 0.05***	2.8 ± 0.13***	3.4 ± 0.02***	2.9 ± 0.02***	1.8 ± 0.05***	2.3 ± 0.11***	2.8 ± 0.13***	2.5 ± 0.12***
RUNX2	1.0 ± 0.05	1.6 ± 0.03***	2.1 ± 0.05***	2.7 ± 0.03***	2.2 ± 0.07***	1.3 ± 0.06***	1.8 ± 0.04***	2.1 ± 0.05***	1.8 ± 0.03***

^*^p < 0.05; ^**^p < 0.01; ^***^p < 0.001, against control.

**Table 2:** Genes that were up-regulated 2.5–2.9 fold for at least one experimental condition/time point.

Gene	DAY1					DAY 7					DAY 14				
	CT	H30	H100	V30	V100	CT	H30	H100	V30	V100	CT	H30	H100	V30	V100
ATF4	1.0	1.5	2.0	1.2	1.5	1.0	1.7	2.3	1.3	1.6	1.0	2.0	2.9	1.5	1.9
BMP4	1.0	1.8	1.9	1.6	1.8	1.0	1.9	2.3	1.7	1.9	1.0	2.1	3.0	1.9	2.1
CACNA1C	1.0	1.5	1.7	1.4	1.5	1.0	1.7	2.1	1.5	1.7	1.0	2.0	2.8	1.7	2.0
CD44	1.0	1.5	1.9	1.2	1.6	1.0	1.8	2.3	1.3	1.8	1.0	2.1	2.9	1.5	2.2
CDC42	1.0	1.7	1.8	1.3	1.7	1.0	1.9	2.3	1.4	1.9	1.0	2.2	2.8	1.6	2.2
CTNNB1	1.0	1.6	2.0	1.3	1.6	1.0	1.7	2.3	1.3	1.6	1.0	1.8	2.7	1.4	1.8
FGF2	1.0	1.7	2.0	1.5	1.7	1.0	1.9	2.2	1.6	2.0	1.0	2.2	2.6	1.8	2.2
SUN2	1.0	1.7	2.1	1.2	1.7	1.0	1.7	2.3	1.2	1.7	1.0	1.9	2.8	1.3	2.0
VEGFA	1.0	2.0	2.1	1.6	2.0	1.0	2.1	2.3	1.7	2.1	1.0	2.2	2.6	1.8	2.3
WNT1	1.0	1.8	2.1	1.5	1.8	1.0	1.9	2.3	1.7	1.9	1.0	2.0	2.7	1.8	2.0
WNT10A	1.0	2.0	2.3	1.7	2.0	1.0	2.1	2.5	1.8	2.1	1.0	2.2	2.7	1.9	2.3

**Table 3:** Genes that were up-regulated 2.0-2.4 fold for at least one experimental condition/time point.

Gene	DAY1					DAY 7					DAY 14				
	CT	H30	H100	V30	V100	CT	H30	H100	V30	V100	CT	H30	H100	V30	V100
BMPR2	1.0	1.8	1.9	1.5	1.8	1.0	1.9	2.1	1.6	1.9	1.0	2.1	2.4	1.7	2.1
BMP7	1.0	1.5	1.8	1.3	1.5	1.0	1.7	2.0	1.5	1.7	1.0	2.0	2.4	1.7	2.0
BGLAP	1.0	1.5	1.7	1.3	1.5	1.0	1.7	2.0	1.5	1.7	1.0	1.9	2.4	1.6	2.0
BMPR1A	1.0	1.5	1.7	1.3	1.5	1.0	1.7	1.9	1.5	1.7	1.0	1.9	2.3	1.7	1.9
CACNA1H	1.0	1.5	1.8	1.3	1.6	1.0	1.7	2.0	1.3	1.7	1.0	1.9	2.2	1.5	1.9
CREB1	1.0	1.2	1.5	1.2	1.3	1.0	1.4	1.8	1.2	1.5	1.0	1.6	2.1	1.3	1.8
CREBBP	1.0	1.2	1.5	1.2	1.3	1.0	1.4	1.7	1.3	1.5	1.0	1.6	2.0	1.4	1.7
ECM1	1.0	1.3	1.6	1.1	1.3	1.0	1.3	1.8	1.2	1.4	1.0	1.5	2.2	1.2	1.6
FGF1	1.0	1.5	1.8	1.2	1.6	1.0	1.7	2.0	1.3	1.7	1.0	1.9	2.2	1.5	1.9
FN1	1.0	1.5	1.9	1.2	1.3	1.0	1.6	2.1	1.4	1.5	1.0	1.7	2.4	1.5	1.6
LAMA1	1.0	1.5	1.6	1.3	1.5	1.0	1.7	1.9	1.4	1.8	1.0	1.9	2.4	1.6	2.0
LRFN5	1.0	1.5	1.8	1.2	1.5	1.0	1.7	2.0	1.4	1.7	1.0	2.0	2.3	1.6	2.0
FOXO1	1.0	1.5	1.7	1.3	1.5	1.0	1.7	2.0	1.5	1.8	1.0	2.0	2.3	1.7	2.0
FRZB	1.0	1.5	1.8	1.3	1.5	1.0	1.7	2.0	1.4	1.7	1.0	1.9	2.3	1.5	1.9
FZD1	1.0	1.6	1.7	1.4	1.6	1.0	1.7	1.9	1.5	1.8	1.0	1.9	2.2	1.6	2.0
GJA1	1.0	1.5	1.8	1.2	1.5	1.0	1.7	2.0	1.3	1.7	1.0	1.9	2.3	1.5	2.0
HAS1	1.0	1.5	1.7	1.3	1.5	1.0	1.7	1.9	1.4	1.7	1.0	1.8	2.1	1.6	1.8
OTX2	1.0	1.6	1.7	1.5	1.6	1.0	1.7	1.9	1.6	1.7	1.0	1.8	2.1	1.6	1.9
PAK1	1.0	1.5	1.6	1.2	1.5	1.0	1.6	1.8	1.3	1.6	1.0	1.7	2.0	1.4	1.8
PTK2	1.0	1.5	1.7	1.3	1.5	1.0	1.7	1.9	1.5	1.7	1.0	1.8	2.1	1.5	1.9
RAC1	1.0	1.5	1.6	1.3	1.5	1.0	1.7	1.8	1.5	1.7	1.0	1.9	2.1	1.6	1.9
RHOA	1.0	1.5	1.8	1.4	1.5	1.0	1.7	2.0	1.5	1.7	1.0	1.9	2.3	1.7	2.0
ROCK1	1.0	1.5	1.8	1.3	1.6	1.0	1.7	2.0	1.4	1.8	1.0	1.9	2.3	1.6	2.0
SYNE1	1.0	1.7	2.0	1.3	1.7	1.0	1.8	2.1	1.4	1.8	1.0	1.9	2.3	1.4	2.0
SUN1	1.0	1.5	1.8	1.2	1.5	1.0	1.6	2.0	1.3	1.7	1.0	1.8	2.2	1.4	1.9
TGFB1	1.0	1.8	2.0	1.5	1.8	1.0	1.8	2.0	1.5	1.8	1.0	1.8	2.0	1.7	1.8
TLN1	1.0	1.5	1.7	1.3	1.5	1.0	1.7	1.9	1.5	1.7	1.0	1.9	2.3	1.6	2.0
TUBB3	1.0	1.5	1.7	1.3	1.5	1.0	1.7	1.9	1.4	1.7	1.0	1.9	2.2	1.6	1.9
TWIST1	1.0	1.5	1.8	1.3	1.6	1.0	1.7	2.1	1.5	1.8	1.0	2.0	2.4	1.7	2.0
WNT3	1.0	1.8	2.0	1.5	1.8	1.0	1.9	2.2	1.6	2.0	1.0	2.1	2.4	1.8	2.1

**Table 4:** Protein (flow cytometry) and transcriptional (RT-PCR) levels of Cadherin-1 (Cdh11), Nesprin-2 (Syne-2), and α-actinin (Actn1) examined on Day 1.

	Control		Horizontal		Vertical	
	*Protein*	*Transcript*	*Protein*	*Transcript*	*Protein*	*Transcript*
Cdh11	52.5 ± 1.0	1.0 ± 0.5	69.9 ± 0.7***	2.6 ± 0.4***	57.5 ± 0.7***	2.6 ± 0.4***
Actn1	57.8 ± 0.6	1.0 ± 0.4	89.9 ± 0.7***	3.3 ± 0.4***	71.7 ± 1.2***	3.3 ± 0.4***
Syne-2	53.1 ± 1.6	1.0 ± 0.4	73.7 ± 0.6***	2.5 ± 0.3***	62.6 ± 1.4***	2.5 ± 0.3***
LaminA/C	—	1.0 ± 0.05	—	2.1 ± 0.1***	—	1.7 ± 0.1***

^*^p < 0.05; ^**^p < 0.01; ^***^p < 0.001, against control.
