# Supplementary material for: Cytoskeletal Configuration Modulates Mechanically Induced Changes in Mesenchymal Stem Cell Osteogenesis, Morphology, and Stiffness
Source: Sci Rep. 2016 Oct 6;6:34791. doi: 10.1038/srep34791 (PMC5052530; doi:10.1038/srep34791)
Supplement: Supplementary Information [file srep34791-s1.pdf]

Supplementary Information

Cytoskeletal Configuration Modulates Mechanically Induced Changes in Mesenchymal Stem Cell Osteogenesis, Morphology, and Stiffness

*Suphannee Pongkitwitoon[1], *Gunes Uzer[2], Janet Rubin[2], Stefan Judex[1]
*Contributed equally
[1]Department of Biomedical Engineering, Stony Brook University
[2]Department of Medicine, UNC Chapel Hill

Corresponding author:
Stefan Judex, Ph.D.
Professor of Biomedical Engineering
Bioengineering Building, Rm 213
Stony Brook University
Stony Brook, NY 11794-5281
Voice: 631-632-1549
Fax: 631-632-8577
Email: stefan.judex@stonybrook.edu

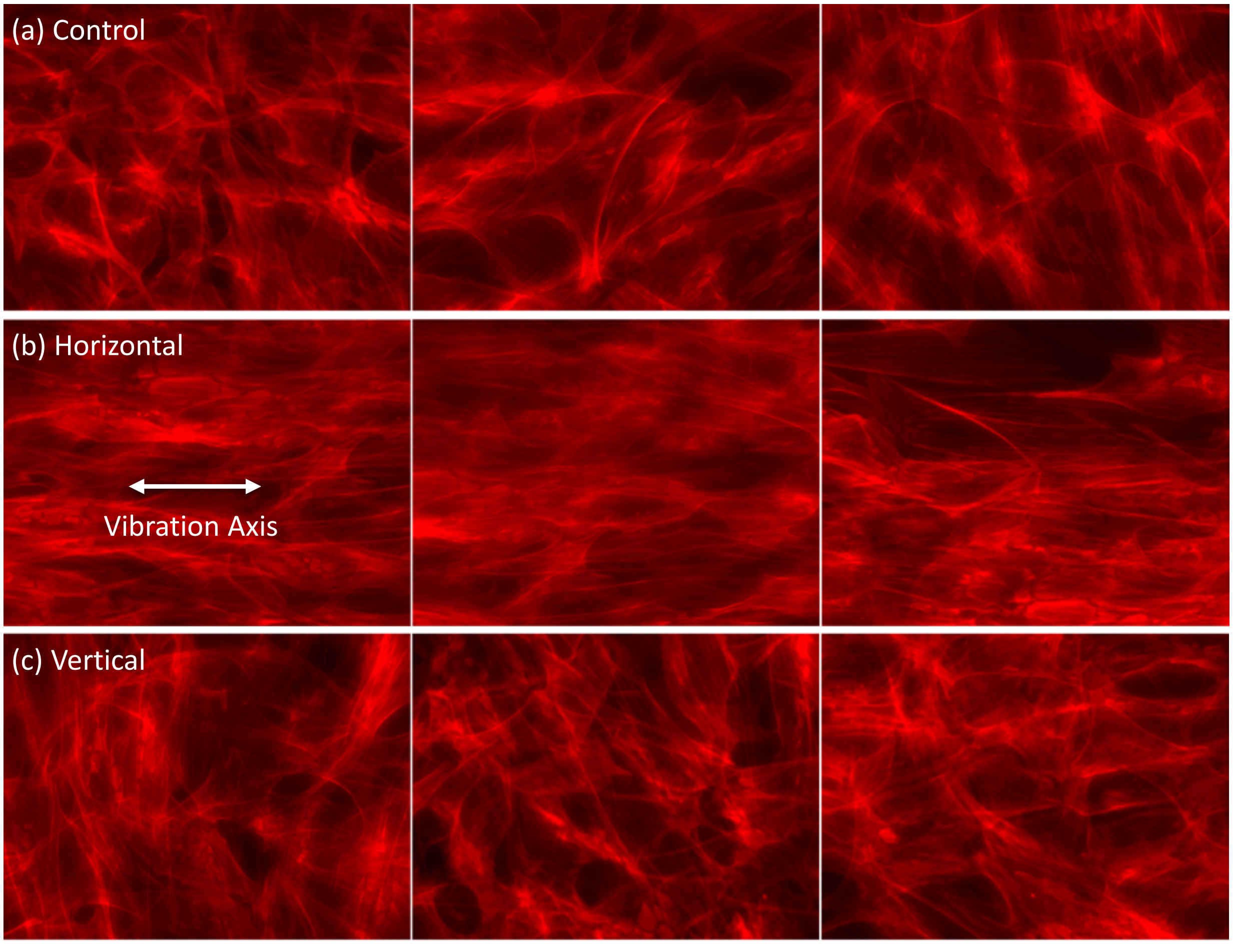

Supplementary Figure 1. Full-field two-photon confocal microscopic fluorescent images of cytoskeletal fibers after exposure to (**a**) non-LIV, (**b**) horizontal LIV (100Hz-0.15g), or (**c**) vertical LIV (100Hz-0.15g).

Supplementary Table 1: List of genes used for PCR array

Unigene	GeneBank	Symbol	Description/Name
Hs.509765	NM_001102	ACTN1	Actinin, alpha 1
Hs.31130	NM_003273	TM7SF2	Transmembrane 7 superfamily member 2 /(Ang1)
Hs.75431	NM_000478	ALPL	Alkaline phosphatase, liver/bone/kidney
Hs.80485	NM_004797	Adipoq	Adiponectin, C1Q
Hs.496487	NM_001675	ATF4	Activating transcription factor 4 (tax-responsive enhancer element B67)
Hs.73853	NM_001200	BMP2	Bone morphogenetic protein 2
Hs.68879	NM_130851	BMP4	Bone morphogenetic protein 4
Hs.471119	NM_001204	BMPR2	Bone morphogenetic protein receptor, type II (serine/threonine kinase)
Hs.473163	NM_001719	BMP7	Bone morphogenetic protein 7 /(OP-1)
Hs.654541	NM_199173	BGLAP	Bone gamma-carboxyglutamate (gla) protein
Hs.524477	NM_004329	BMPR1A	Bone morphogenetic protein receptor, type IA
Hs.459642	NM_021098	CACNA1Hb	Calcium channel, voltage-dependent, T type, alpha 1H subunit
Hs.118262	NM_000719	CACNA1C	Calcium channel, voltage-dependent, L type, alpha 1C subunit
Hs.502328	NM_000610	CD44	CD44 molecule
Hs.690198	NM_001791	CDC42	Cell division cycle 42 (GTP binding protein, 25kDa)
Hs.116471	NM_001797	CDH11	Cadherin 11, type 2, OB-cadherin (osteoblast)
Hs.172928	NM_000088	COL1A1	Collagen, type I, alpha 1
Hs.516646	NM_004379	CREB1	CAMP responsive element binding protein 1
Hs.459759	NM_004380	CREBBP	CREB binding protein
Hs.591402	NM_000757	CSF1	Colony stimulating factor 1
Hs.476018	NM_001904	CTNNB1	Catenin (cadherin-associated protein), beta 1, 88kDa
Hs.81071	NM_004425	ECM1	Extracellular matrix protein 1
Hs.391561	NM_001442	Fabp4	Fatty acid binding protein 4, adipocyte
Hs.483635	NM_000800	FGF1	Fibroblast growth factor 1 (acidic)
Hs.284244	NM_002006	FGF2	Fibroblast growth factor 2 (basic)
Hs.287370	NM_020638	FGF23	Fibroblast growth factor 23
Hs.203717	NM_002026	FN1	Fibronectin 1
Hs.136893	NM_152447	LRFN5	Fibronectin type 3 domain containing protein 5 (FNDC5, Irisin)
Hs.370666	NM_002015	FOXO1	Forkhead box O1
Hs.128453	NM_001463	FRZB	Frizzled-related protein
Hs.94234	NM_003505	FZD1	Frizzled family receptor 1
Hs.74471	NM_000165	GJA1	Gap junction protein, alpha 1, 43kDa, /(GJAL)
Hs.2171	NM_004962	GDF10	Growth differentiation factor 10
Hs.196384	NM_000963	PTGS2	Prostaglandin-endoperoxide synthase 2 (and cyclooxygenase), /(COX2)
Hs.655209	NM_004723	ARHGEF2	Rho/rac guanine nucleotide exchange factor (GEF) 2, /GEF-H1
Hs.57697	NM_001523	HAS1	Hyaluronan synthase 1

Hs.518726	NM_004967	IBSP	Integrin-binding sialoprotein, /(BSP)
Hs.643447	NM_000201	ICAM1	Intercellular adhesion molecule 1
Hs.160562	NM_000618	IGF1	Insulin-like growth factor 1 (somatomedin C)
Hs.643120	NM_000875	IGF1R	Insulin-like growth factor 1 receptor
Hs.654504	NM_002181	IHH	Indian hedgehog /(HHG2)
Hs.436873	NM_002210	ITGav	Integrin, alpha V (vitronectin receptor, alpha polypeptide, antigen CD51)
Hs.505654	NM_002205	ITGa5	Integrin, alpha 5 (fibronectin receptor, alpha polypeptide)
Hs.218040	NM_000212	ITGb3	Integrin, beta 3 (platelet glycoprotein IIIa, antigen CD61)
Hs.270364	NM_005559	LAMA1	Laminin, alpha 1
Hs.6347	NM_002335	LRP5	Low density lipoprotein receptor-related protein 5
Hs.178023	NM_005593	Myf5	Myogenic factor 5
Hs.469880	XM_940482	LOC389031	Myosin
Hs.16355	NM_005964	MYH10	Myosin, heavy chain 10, non-muscle
Hs.25155	NM_005863	NET1	Neuroepithelial cell transforming 1
Hs.495473	NM_017617	NOTCH1	Notch 1
Hs.592227	NM_007052	NOX1	NADPH oxidase 1
Hs.288655	NM_021728	OTX2	Orthodenticle homeobox 2
Hs.435714	NM_002576	PAK1	P21 protein (Cdc42/Rac)-activated kinase 1
Hs.458573	NM_006207	PDGFRL	Platelet-derived growth factor receptor-like
Hs.553498	NM_006218	PIK3CA	Phosphoinositide-3-kinase, catalytic, alpha polypeptide
Hs.696032	NM_006238	PPARD	Peroxisome proliferator-activated receptor delta, PPARb
Hs.162646	NM_015869	PPARg	Peroxisome proliferator-activated receptor gamma
Hs.500466	NM_000314	PTEN	Phosphatase and tensin homolog
Hs.395482	NM_005607	PTK2	PTK2 protein tyrosine kinase 2 /(FAK)
Hs.413812	NM_006908	RAC1	Ras-related C3 botulinum toxin substrate 1 (rho family, small GTP binding protein Rac1)
Hs.247077	NM_001664	RHOA	Ras homolog gene family, member A
Hs.306307	NM_005406	ROCK1	Rho-associated, coiled-coil containing protein kinase 1
Hs.535845	NM_004348	RUNX2	Runt-related transcription factor 2
Hs.135015	NM_001038633	RSPO1	R-spondin 1
Hs.209402	NM_152860	SP7	Sp7 transcription factor /(Osterix)
Hs.313	NM_000582	SPP1	Secreted phosphoprotein 1, /(Osteopontin, OPN)
Hs.349204	NM_025237	SOST	Sclerostin
Hs.596449	NM_001235	SERPINH1	Serpin peptidase inhibitor, clade H (heat shock protein 47), member 1, (collagen binding protein 1)
Hs.12967	NM_182961	SYNE1	Spectrin repeat containing, nuclear envelope 1 /(MYNE1, human)
Hs. 525392	NM_182914	SYNE2	Spectrin repeat containing, nuclear envelope 2 /(Nesprin2)
Hs.604588	NM_005900	SMAD1	SMAD family member 1
Hs.75862	NM_005359	SMAD4	SMAD family member 4
Hs.647409	NM_000346	SOX9	SRY (sex determining region Y)-box 9

Hs.438072	NM_025154	sun1	Sad1 and UNC84 domain containing 1
Hs.517622	NM_015374	sun2	Sad1 and UNC84 domain containing 2
Hs.463059	NM_003150	STAT3	Signal transducer and activator of transcription 3 (acute-phase response factor)
Hs.645227	NM_000660	TGFB1	Transforming growth factor, beta 1
Hs.471014	NM_006289	TLN1	Talin 1
Hs.241570	NM_000594	TNF	Tumor necrosis factor
Hs.333791	NM_003701	TNFSF11	Tumor necrosis factor (ligand) superfamily, member 11 /(RANKL)
Hs.591983	NM_004620	TRAF6	TNF receptor-associated factor 6
Hs.654708	NM_147686	TRAF3IP2	TRAF3 interacting protein 2 /(ACT1)
Hs.511743	NM_006086	TUBB3	Tubulin, beta 3
Hs.66744	NM_000474	TWIST1	Twist homolog 1
Hs.249211	NM_021833	UCP1	Uncoupling protein 1 (mitochondrial, proton carrier)
Hs.73793	NM_003376	VEGFA	Vascular endothelial growth factor A
Hs.248164	NM_005430	WNT1	Wingless-type MMTV integration site family, member 1
Hs.445884	NM_030753	WNT3	Wingless-type MMTV integration site family, member 3
Hs.121540	NM_025216	WNT10A	Wingless-type MMTV integration site family, member 10A
Hs.377360	NM_001080435	WHAMM	WAS protein homolog associated with actin, golgi membranes and microtubules
Hs.520640	NM_001101	ACTB	Actin, beta
Hs.592355	NM_002046	GAPDH	Glyceraldehyde-3-phosphate dehydrogenase
Hs.546285	NM_001002	RPLP0	Ribosomal protein, large, P0
Hs.534255	NM_004048	B2M	Beta-2-microglobulin
Hs.412707	NM_000194	HPRT1	Hypoxanthine phosphoribosyltransferase 1
